# Determination of aluminum concentrations in biological specimens: application in the clinical laboratory

**DOI:** 10.1515/almed-2022-0056

**Published:** 2022-06-22

**Authors:** Sonia Pérez San Martín, Josep Miquel Bauçà, Eduardo Martinez-Morillo

**Affiliations:** Servicio de Análisis Clínicos, Hospital Universitario de Cabueñes, Gijón, Spain; Comisión de Elementos traza, Sociedad Española de Medicina de Laboratorio, Barcelona, Spain; Servicio de Análisis Clínicos, Hospital Universitari Son Espases, Palma de Mallorca, Spain; Servicio de Análisis Clínicos y Bioquímica Clínica, Complejo Asistencial Universitario de Salamanca, Salamanca, Spain

**Keywords:** atomic absorption, inductively coupled plasma mass spectrometry (ICP-MS), toxic metals, toxicity, trace elements

## Abstract

Aluminum enters the body primarily through diet or occupational exposure, and is cleared through urine. However, this trace element may accumulate and cause toxicity in subjects with renal insufficiency, and even in dialysis patients. The mechanism of aluminum toxicity is related to increased oxidative and inflammatory stress, iron and calcium dyshomeostasis, or cholinergic dysregulation, among other. A review was conducted on the specimens and analytical methods used to determine aluminum in biological specimens and dialysis water. This paper describes the most relevant aspects related to quality assurance. This is a practical guideline for the development and implementation of a reliable method for determination of aluminum in the clinical laboratory. Serum aluminum is the main biomarker of toxicity. For cases of chronic exposure, urine testing is recommended. At present, inductively coupled plasma mass spectrometry (ICP-MS) is the gold-standard determination method, since it has been proven to have the best quantification limits, selectivity and robustness. Clear recommendations are provided in relation to the specimens used for aluminum determination. Relevant pre-analytical, analytical, and post-analytical considerations are also presented.

## Introduction

Aluminum is the most abundant metal in the Earth’s crust; however, it has not been found to play any biological role in humans. The World Health Organization (WHO) established the acceptable daily intake of aluminum at 1 mg/kg of body mass [1]. This metal, however, is widely used in the industry and as an additive in drinking water, processed food, baby and parenteral formula, deodorants, and medicines.

Exposure to high concentrations of aluminum through food and, to a lesser extent, the environment, along with occupational exposure may have deleterious effects on human health. The main route for entry of aluminum in the body is by inhalation [2, 3].

Although there are significant variations in total aluminum intake depending on the place of residence and diet composition, about 10 mg of aluminum enter the human body daily [4]. In blood, 80% of aluminum is bound to transferrin and rapidly excreted by the kidneys [4, 5]. Aluminum may accumulate in the body of patients exposed to high amounts of aluminum or with clearance deficiencies [6], such as patients with chronic kidney disease (CKD) or hemodialysis patients, as a result of dialysis fluid contamination or intake of aluminum-containing chelating agents and antiacids. Some decades ago, the main complication of CKD in dialysis patients was dialytic encephalopathy associated with osteomalacia and anemia [7, 8]. The recent guideline *Kidney Disease: Improving Global Outcomes* (KDIGO), recommends avoiding long-term use of aluminum-based phosphate chelators in patients with G3-G5D CKD and prevent dialysis fluid contamination with aluminum, especially in G5D category patients [9].

Likewise, the Spanish Society of Nephrology (SEN) recommends that levels of aluminum in blood should be maintained below 20 μg/L (0.74 μmol/L) in patients receiving hemodialysis [10]**.** However, aluminum intoxication in dialysis patients has a low prevalence, and, according to some authors, monitoring aluminum concentrations in the majority of dialysis patients is not cost-effective, while other authors recommend monitoring aluminum only in patients with risk factors [7, 11].

The solubility of aluminum compounds determines its toxicokinetics and health risks [2]. Aluminum primarily accumulates in the bones, thereby hindering calcium exchange and limiting bone resorption dependent on parathyroid hormone (PTH) and 1,25-dihydroxyvitamin D, and reducing osteoblast activity [12]. In addition, high aluminum concentrations are also found in patients with celiac disease, hemochromatosis, sickle cell anemia, and exostosis, as well as in patients with knee or hip replacements [13]. Aggregation of aluminum in bone tissue may be especially relevant in children and adolescents, with severe clinical effects [14].

Elevated levels of aluminum have been recently observed in brain tissue of patients with Alzheimer’s disease, autism, and epilepsy [1, 15]. Aluminum induces brain damage and behavior alterations through not clearly understood mechanisms, including apoptosis, phosphorylation of tau protein, β-amyloid protein accumulation, formation of reactive oxygen species, neuronal cell necrosis, metal dyshomeostasis, and changes in the antioxidant defense system [16, 17], which causes apoptosis in neuron and glial cells [18] and triggers a pro-inflammatory state, associated with an increase in vascular events [17].

In a meta-analysis, Wang et al. [19], concluded that chronic exposure to aluminum is associated with a higher risk of developing Alzheimer’s disease. In the same line, Colomina et al. [20] obtained consistent results confirming this association, although aluminum concentrations, time of exposure or other associated risk factors have not yet been determined.

With respect to the use of aluminum in vaccines, some authors associate it with persistent myalgia, fatigue or autoimmune diseases, although there is no solid evidence of the association of aluminum with these diseases [21]. Aluminum salts have been used as adjuvants for decades to increase immune effect and ensure effectiveness. The Centers for Disease Control and Prevention (CDC) consider that vaccines only contain a low amount of aluminum that is scarcely absorbed by the body [22], with minimal adverse reactions at the puncture site.

At present, aluminum is tested in the clinical laboratory primarily to monitor dialysis patients and water, and to control metallic implant wear.

## Purpose and scope

The purpose of this document is to review the laboratory methods used to determine aluminum concentrations in biological specimens, assess its physiopathological value, and provide a practical guideline for the implementation of this technology in the clinical laboratory. This method can be applied in clinical laboratories fitted with facilities and equipment for the determination of trace elements and toxic metals.

## Biological specimens for determination

### General considerations

Contamination is the main problem in trace elements determination, including aluminum. Specimens must be carefully collected and handled. The *Clinical and Laboratory Standards Institute* (CLSI) guideline provides a description of pre-analytical procedures [23].

The Spanish Society of Laboratory Medicine (SEQC^ML^) published the technical and environment requirements and safety measures to be adopted by clinical laboratories for determination of trace elements [24, 25]. In addition, it is necessary to use standards and reagents with a high level of purity, as well as ultrapure type 1 water [23].

### Blood

Determination of serum concentrations is useful to establish a possible intoxication. It is the method of choice for routine screening and monitoring of metallic implant wear [3, 26, 27].

The majority of the tubes and tools used in blood collection contain rubber made of aluminum silicate. It is recommended to collect serum in specific vacuum tubes for trace elements. Separating gel vacuum tubes are allowed, but usage of glass tubes should be avoided [23]. It is responsibility of the laboratory to ensure the interchangeability of results, in accordance with quality specifications.

The laboratory must ensure that the material in contact with the specimen (assay tubes, pipette tips, containers, etc.) does not contain aluminum, thereby posing a risk of contamination. Otherwise, material must be previously decontaminated by washing it in nitric acid 10% for 12–24 h and repeatedly irrigated with ultrapure water before use [23].

Once blood has been collected and coagulated, it is centrifuged for 10 min at 1,000–1,200 g in a closed container to prevent contamination from centrifugation or evaporation [23]. Serum is stored in adequately sealed polypropylene or polystyrene tubes for 14 days at 4 °C, or frozen for later analysis [28].

### Urine

Urine is the sample of choice for monitoring chronic exposure to aluminum and assesses chronic exposure in cases of occupational or environment exposure, as it has a higher sensitivity [3, 26, 27].

In workers exposed to aluminum, urine concentrations 1 or 2 days after exposure is a reliable indicator of aluminum concentrations in the body [27].

24 h urine is more suitable for aluminum determination. For a correct determination of aluminum in 24 h urine, it is very important that an accurate diuresis value is obtained. As it occurs with serum, it is recommended to screen for potential sources of contamination of the material used for urine analysis; otherwise, it must be previously washed with acid. To preserve sample stability, it can be acidified upon reception in the laboratory [23]. It is essential to avoid errors during 24 h urine collection and prevent contamination from containers with exogenous aluminum.

Once samples are homogenized, aliquots are distributed in clean tubes and refrigerated or frozen for later analysis. If the sample is frozen, thawing is followed by homogenization and centrifugation.

### Bone

Bone samples are collected from the iliac crest using a trocar, by intraoperative biopsy or *post mortem*, in the case of samples collected to evaluate aluminum concentrations in the population of reference [29]. The sample is stored in a polypropylene tube at −20 °C for post-digestion processing. Bone material remains stable for three months when stored at −20 °C and for one year when lyophilized [28].

### Dialysis water and fluid

The same recommendations are applicable in terms of sample collection and handling as for serum and urine [28]. Water samples are stored in sterile tubes such as those used for serum, or in a urine tube, in case additional determinations are being performed and a higher volume is required.

The SEN guideline sets the maximum aluminum concentration at 10 μg/L (0.37 μmol/L) in ultrapure water for hemodialysis, with biannual controls [10].

Determination of aluminum in hair, nails, or other biological fluids is out of the scope of these recommendations [30], [31], [32].

## Determination methods

There is a wide range of laboratory techniques available to determine aluminum concentrations. However, some of these techniques are no longer used, due to their insufficient detection limits or significant number of interferences. Such is the case of UV–visible absorption spectroscopy, fluorescence spectroscopy, flame atomic absorption spectrometry, X-ray fluorescence spectrometry, and neutron activation analysis. These techniques are out of the scope of this study.

### Electrothermal atomic absorption spectrometry

In clinical laboratories, electrothermal atomic absorption spectrometry (ETAAS) has been the technique of choice due to its simplicity. In addition, it does not require sample pretreatment and a small sample volume is necessary. The detection limit is around 1–2 μg/L (0.04–0.07 μmol/L) [28].

This technique requires the use of background correctors, being Zeeman correction the method of choice, which prevents molecular interferences and confers a high specificity. Matrix modifiers are also used which allow chlorine atoms displacement and prevent the formation of volatile aluminum chloride in steps prior to atomization. In practice, specimens are diluted in water, nitric acid and a surfactant, such as Triton X-100^®^. Magnesium nitrate is often used as a modifier to reduce the volatility of aluminum chloride. However, matrix modifiers are a source of contamination and its use is not recommended [28].

The methods for determination of aluminum concentrations in different types of specimens by ETAAS are described in the Supplementary Material.

### Inductively coupled plasma mass spectrometry

The use of this technique for determination of trace elements and toxic metals in biological matrices has grown exponentially, despite the complexity and high cost of the analyzer. According to the quality assurance program of the *Occupational and Environmental Laboratory Medicine* (OELM) for serum, inductively coupled plasma mass spectrometry (ICP-MS) was the most widely used technique in Europe in 2021.

Enhanced detection limits and selectivity, the possibility of performing multi-element analysis, and simplicity of sample preparation are some of the advantages [33], being the technique of choice when multiple trace elements are to be analyzed in a high number of samples [34].

The high temperatures that plasma undergoes (6,000–10,000 K), the stability and environment of chemically inert argon eliminate the numerous interferences found in other types of atomic spectrometry, with detection limits reaching 0.1–1 μg/L (0.004–0.04 μmol/L) for most of the elements of the periodic table. Therefore, ICP-MS is the technique of choice for determination of aluminum in biological specimens and dialysis water.

Given the high sensitivity of the analyzer, and since only a minimal variation in sample preparation or insertion may dramatically change instrumental response, it is recommended to use internal standards, a known amount of a compound other than the analyte and absent in the sample, which is added to correct the typical fluctuations of instrumental response [33].

There are two types of interferences in ICP-MS [27, 33]:

– Spectroscopic, which occur when an ionic species has the same mass/charge (*m*/*z*) ratio as the analyte. These interferences originate from polyatomic ions and refractory oxide ions. To avoid these interferences, an isotope other than the trace element is selected, with a *m*/*z* ratio different to that of the interfering substance. Another method to prevent interferences is by using a collision reaction cell with an inert gas (kinetic elimination) or reagent (chemical elimination).
– Non-spectroscopic: these interferences are caused by the matrix, which causes a change of signal due to an alteration in sample transportation, level of ionization of the analyte in the source of plasma, or in the level of extraction of ions. They can be corrected by diluting the sample following separation of the interfering compounds; by inserting the sample in a different way (by electrothermal vaporization, using a nebulizer at 2 °C, by flow injection); or using an internal standard. In some cases, analyte quantification can be performed using the standard addition method.

#### Determination of aluminum concentration in different types of specimens by ICP-MS

##### Analytical conditions

Analytical conditions depend on the analyzer used. The aluminum-27 isotope (the only natural stable isotope of this element) is monitored using scandium (Sc) as an internal standard, which has an atomic mass of 44.95.

##### Sample preparation

The low quantification limits of this technology enable higher sample dilutions. Satisfactory results are obtained using dilutions close to 1/14 or 1/18 for serum and 1/9 for urine with 0.5% HNO_3_ for standards and controls. Based on the pipetting volume of the analyzer, it is recommended to use a 1/14 dilution by mixing 250 µL of the sample with 3,250 µL of 0.5% HNO_3_ as a diluent.

##### Standard preparation

From a certified standard or a primary standard solution of 1 g/L of Al (37.03 mmol/L), calibration curve patterns are obtained using an aqueous storage solution of 100 mg/L (3.7 mmol/L) prepared by serial dilutions. Given that serum aluminum concentrations above 60 μg/L (2.22 μmol/L) suggest a possible contamination and are rarely found [27], it is not necessary that the calibration line exceeds that value. If the sample is diluted 1/4, the line can be done using calibrators with concentrations within the range of 0.5 and 4.5 μg/L, which enables quantification of samples with aluminum concentrations of 7–63 μg/L (0.26–2.33 μmol/L). Otherwise, dilution of the sample 1/6 or lower enables quantification of samples with concentrations above 3 μg/L (0.11 μmol/L).

As it occurs with other laboratory technologies, if matrix effect is detected during aluminum quantification, standard addition is recommended.

### Quality assurance

#### Laboratory quality standards based on biological variability

Since aluminum is a toxic element without a known biological role, biological variability criteria cannot be used to establish analytical quality specifications, since the available data available are very limited [35].

Taylor et al. recommend a total error for aluminum below 5 μg/L (or <20%) of the target concentration, which is the desirable laboratory quality goal [36].

#### Control materials

The incorporation of reference materials of reference to the analytical process is essential for quality assessment and plays a key role in the traceability of determinations. In routine practice, laboratories use internal quality controls with a similar or the same matrix to that of the specimens to be analyzed (serum, blood, and urine) to ensure the reliability of results. Therefore, serum controls Lyphochek^®^ Whole Blood Metals Quality Control (BIO-RAD, QCNetT™), Seronorm Trace Elements Serum (Seronorm™ Trace Element Serum [Nycomed Pharma AS, Noruega]), ClinChek^®^ Serum Controls, lyophilised for Trace Elements (RECIPE^®^), or others are recommended.

The following urine samples are recommended: Lyphochek^®^ Urine Metals Control (BIO-RAD), Seronorm Trace Elements Urine (Nycomed), ClinChek^®^ Urine Controls, lyophilised for Trace Elements (RECIPE^®^), or other similar.

#### External quality assessment schemes

To assess the trueness, accuracy and reproducibility of results, benchmarking based on external quality assurance schemes (EQA) is performed. In addition, it is a requirement of ENAC for clinical laboratories to be granted UNE-EN ISO 15189:2013 certification.

There are multiple specific external quality assurance schemes for trace elements, including aluminum.

The SEQC^ML^, in collaboration with other countries, participates in the organization of the OELM External Quality Program, by sharing samples, their database, and report formats adopting a federal approach. Nevertheless, they remain being independent organizations that are engaged in the program as independent organizations, and use their own language, hold independent user meetings and offer other assistance systems (http://www.trace-elements.eu). Matrices include serum, whole blood, and urine.

Other relevant programs include the UKNEQAS for Trace Elements (TEQAS, United kingdom), which uses serum, blood, urine, and dialysis water and fluids, and the QMEQAS of the *Centre de Toxicologie du Québec* (Canada), which uses serum, blood, urine, and hair as the matrix.

## Reference intervals

Aluminum concentrations may vary significantly based on the geographical region, dietary habits, occupational environment, lifestyle, and genetic variations [27, 37].

In Australia, a recent study in 120 healthy adults revealed mean plasma aluminum concentrations of 6.9 μg/L, as measured by ICP-MS, without significant age- or sex-based differences [37]. In the same line, the French study IMEPOGE in 2000 healthy subjects uncovered a mean aluminum concentration of 4.32 μg/L, with significantly higher values in men, as compared to women [38]. In another two studies in Belgium and Canada, the authors report median values of aluminum concentrations below the detection limit of detection of the technique (3.1 and 8 μg/L, respectively) [39, 40].

With respect to urine aluminum concentrations, the French study documented mean values of 4.25 μg/L or 3.99 μg/g creatinine [38], whereas a study in Belgium revealed median aluminum concentrations of 2.17 μg/L or 2.04 μg/g creatinine [41].

The accumulation of aluminum in the bones, which are the primary deposit in case of aluminum overload, the data reported in the literature are inconsistent, with mean values ranging from 1 to 9 μg/g of dry weight [27].

Each laboratory should establish and verify their own reference values using a standard procedure [42] or otherwise verify the values of reference adopted. The following reference values of reference are provided as guidance material to assist laboratories in establishing and regularly reviewing their results [23, 27]:

– Serum: <10 μg/L (<0.37 μmol/L)
– Urine: <7 μg/day (<0.26 µmol/day)
– Tissue: <2 μg/g dry weight (<0.07 μmol/kg)

Additionally, the Mayo Clinic recently published the following reference values of reference for adults [43]:

– Serum: <7 μg/L (0.26 μmol/L), <60 μg/L (2.23 μmol/L) (in dialysis patients).

Patients wearing an aluminum-based prosthetic implant may exhibit concentrations > 6 μg/L. A moderate elevation of concentrations (6–10 μg/L) suggests that the prosthesis is in good state. Aluminum concentrations >10 μg/L (0.37 μmol/L) are suggestive of implant wear.

– 24 h urine: <13 μg/24 h (<0.48 µmol/24 h)
– Spontaneous miction urine: <14 μg/g creatinine (0.52 μmol/g creatinine)

## Conclusions

In this study, a review was performed of the specimens and analytical methods used to determine aluminum in biological specimens and dialysis water. The purpose of this study is to provide basic guidelines to clinical laboratory professionals who need to quantify aluminum. Apart from some pre-analytical considerations, recommendations are provided for analytical quality assurance and for the establishment of reference intervals and interpretation of results.

## Supplementary Material

Supplementary Material
