# Supplementary material for: Determination of aluminum concentrations in biological specimens: application in the clinical laboratory
Source: Adv Lab Med. 2022 Jun 22;3(2):153–9. doi: 10.1515/almed-2022-0056 (PMC10197291; doi:10.1515/almed-2022-0056)
Supplement: Supplementary file 1 — Supplementary Material [file j_almed-2022-0056_suppl.docx]

**ANNEX 1.** Supplementary material:

**Procedure for determination of aluminum concentration in different types of specimens by electrothermal atomic absorption spectrometry.**

#### **Analytical conditions**

A hollow-cathode or mono-element discharge lamp is required for a higher light intensity, a wavelength of 309.3 nm, and a slit of 0.7 nm. Pyrolytic graphite furnace L’Vov platforms are required. In general, the procedures described in the literature establish three drying stages for sample evaporation (Table 1, Table 2). Drying temperatures range from 90ºC to 200 ºC, with mineralization temperatures ranging from 600 to 1500ºC, and atomization temperatures reaching 2600ºC. Table 1 shows a rapid and widely available program for serum/plasma. A program for urine and bone is described in Table 2. However, each user should optimize these conditions according to the equipment used (48, 49). A sample volume of 20 μL is recommended. The peak area reading is used, with an integration time of 2 s. An additional cleaning step at 2650ºC is recommended.

# This technique yields quantification limits near 0.14 µmol/L.

#### **Sample preparation**

To determine aluminum concentrations in serum, it is recommended to dilute serum 1:2 or 1:3 in a 0.2% nitric acid (HNO_3_) matrix modifier solution and Triton X-100®.

Acidified urine is prepared for immediate analysis, centrifuged and diluted 1:2 in a 0.1% nitric acid matrix modifier solution and 0.2% Triton X-100 as a surfactant.

Preparation of the sample for determination of aluminum concentrations in bone is entirely performed in a clean chamber to prevent contamination from atmospheric dust. Eight hours after graphite furnace processing, drying of the bone cylinder is performed (20-30 mg of weight) in the stove at 90ºC until weight remains constant. Then, digestion is performed in teflon 3mL recipients with 200 µL of suprapur nitric acid and stored at room temperature for 1 hour, and later in a stove at 8ºC - 90 ºC for 5 hours. A clear fluid is obtained, which is dissolved to 500 µL with water. This solution is then diluted with water treated by inverse osmosis. Dilutions are dependent on sample aluminum concentration, since in the conditions described, the measure is valid for intervals of 10 to 40 µg/L (0.37 to 1.48 µmol/L).

To measure net water and dialysis fluids, specimens are diluted 1:3 in nitric acid 0.2%. Higher dilutions can be performed when concentrations are elevated.

#### **Standard preparation**

Concentration standards can be prepared at 10, 20 and 40 µg/L (0.37, 0.74 and 1.48 µmol/L) in 0.2% HNO_3_ . A certified standard of 1 g/L in 2% HNO_3_  can be initially used. The blank is only composed of 0.2% HNO_3_  and 0.2% Triton X-100® .

In the case of bone, calibration is carried out by standard additions, which are time-consuming but are the best method to minimize matrix effect, given the high phosphate content of the solution resulting from bone digestion *(*50*)*.

Table 1. Recommended temperature scheme for determination of aluminum in serum by ETAAS

| Stage | Temperature (ºC) | Time (s) | | Argon flow (mL/min) | Reading |
| --- | --- | --- | --- | --- | --- |
|  |  | Initial slope | Final slope |  |  |
| Drying 1 | 90 | 5 | 5 | 300 | No |
| Drying 2 | 130 | 10 | 10 | 300 | No |
| Drying 3 | 200 | 5 | 5 | 300 | No |
| Mineralization 1 | 600 | 1 | 20 | 300 | No |
| Mineralization 2 | 1.500 | 10 | 15 | 300 | No |
| Atomization | 2.600 | 0 | 2 | 0 | Yes |
| Cleaning | 2.650 | 1 | 2 | 300 | No |

Table 2. Recommended temperature scheme for determination of aluminum in urine and bone by ETAAS

| Stage | Temperature (ºC) | Time (s) | | Argon flow (mL/min) | Reading |
| --- | --- | --- | --- | --- | --- |
|  |  | Initial slope | Final slope |  |  |
| Drying 1 | 90 | 5 | 5 | 300 | No |
| Drying 2 | 130 | 5 | 5 | 300 | No |
| Drying 3 | 200 | 2 | 2 | 300 | No |
| Mineralization 1 | 600 | 1 | 20 | 300 | No |
| Mineralization 2 | 1.450 | 2 | 15 | 300 | No |
| Atomization | 2.600 | 0 | 2 | 0 | Yes |
| Cleaning | 2.650 | 1 | 2 | 300 | No |
